# Remaining Useful Life Prediction of Rolling Bearings Based on Multi-scale Permutation Entropy and ISSA-LSTM

**DOI:** 10.3390/e25111477

**Published:** 2023-10-25

**Authors:** Hongju Wang, Xi Zhang, Mingming Ren, Tianhao Xu, Chengkai Lu, Zicheng Zhao

**Affiliations:** School of Mechanical and Electrical Engineering, China University of Mining & Technology (Beijing), Beijing 100083, China

**Keywords:** remaining useful life, maximum correlation kurtosis deconvolution, multi-scale permutation entropy, long short-term memory

## Abstract

The performance of bearings plays a pivotal role in determining the dependability and security of rotating machinery. In intricate systems demanding exceptional reliability and safety, the ability to accurately forecast fault occurrences during operation holds profound significance. Such predictions serve as invaluable guides for crafting well-considered reliability strategies and executing maintenance practices aimed at enhancing reliability. In the real operational life of bearings, fault information often gets submerged within the noise. Furthermore, employing Long Short-Term Memory (LSTM) neural networks for time series prediction necessitates the configuration of appropriate parameters. Manual parameter selection is often a time-consuming process and demands substantial prior knowledge. In order to ensure the reliability of bearing operation, this article investigates the application of three advanced techniques—Maximum Correlation Kurtosis Deconvolution (MCKD), Multi-Scale Permutation Entropy (MPE), and Long Short-Term Memory (LSTM) recurrent neural networks—for the prediction of the remaining useful life (RUL) of rolling bearings. The improved sparrow search algorithm (ISSA) is employed for configuring parameters in the Long Short-Term Memory (LSTM) network. Each technique’s principles, methodologies, and applications are comprehensively reviewed, offering insights into their respective strengths and limitations. Case studies and experimental evaluations are presented to assess their performance in RUL prediction. Findings reveal that MCKD enhances fault signatures, MPE captures complexity, and LSTM excels in modeling temporal patterns. The root mean square error of the prediction results is 0.007. The fusion of these techniques offers a comprehensive approach to RUL prediction, leveraging their unique attributes for more accurate and reliable predictions.

## 1. Introduction

Rolling bearings are crucial components in various industrial systems, including machinery, automotive, aerospace, and wind turbines [1]. The reliable and efficient functioning of these systems heavily depends on the health and performance of rolling bearings. However, the degradation and failure of rolling bearings can lead to costly downtime, productivity losses, and safety risks. To mitigate these issues, the concept of remaining useful life (RUL) prediction has gained significant attention in recent years. RUL prediction aims to estimate the remaining operational lifespan of rolling bearings, enabling proactive maintenance strategies and optimizing asset management.

Traditional maintenance strategies, such as time-based or reactive maintenance, often result in inefficient resource allocation and unnecessary maintenance activities [2]. By accurately predicting the RUL of rolling bearings, maintenance activities can be planned in advance, leading to reduced downtime, optimized maintenance schedules, and cost savings. RUL prediction also enables condition-based maintenance, where maintenance actions are triggered based on the actual health condition of rolling bearings rather than arbitrary time intervals. This approach enhances reliability, minimizes the risk of catastrophic failures, and improves overall system performance.

Rolling bearings’ RUL prediction has traditionally relied on statistical and data-driven methods [3]. However, recent advancements in signal processing, data analytics, and machine learning techniques have provided new opportunities to enhance the accuracy and reliability of RUL prediction. Data-driven remaining useful life (RUL) prediction typically involves three key stages: data acquisition and preprocessing, feature extraction and selection, and degradation behavior modeling and RUL estimation [4].

Presently, the generation of bearing-fault time series involves convolving vibration signals with various noise signals during signal transmission. However, this approach adversely impacts the accuracy of the trained prediction model. To address this issue, the initial phase of this research focuses on preprocessing the original time series data. In this context, Dong et al. combined spectral wavelet transform and detrended fluctuation analysis [5], introducing a non-iterative denoising method tailored for filtering nonlinear vibration signals. Additionally, Yan et al. explored the discrete convolution wavelet transform (DCWT) to decompose and reconstruct signals [6], especially for rapidly changing signal processing. Although significant progress has been made, the wavelet-based functions suffer from limited selection capacity.

Bagheri et al. proposed a dynamic response decomposition approach based on VMD to uncover modal properties in engineering structures [7]. Furthermore, Zhang et al. delved into the fractal properties of vibration signals from rolling element bearings and devised an effective method for assessing and diagnosing bearing defects [8]. Subsequent investigations unveiled the substantial impact of the decomposition mode parameter “K” and the penalty coefficient “η” on decomposition effectiveness, necessitating their careful adjustment to address varying parameters. To mitigate mode mixing issues in complex vibration signals, Zhao X et al. introduced an approach leveraging the single-objective salp swarm algorithm to optimize the penalty coefficient “η” of VMD [9]. Feng et al. employed the whale optimization algorithm (WOA) to optimize VMD parameters, enabling adaptive decomposition and noise reduction in vibration signals [10].

Concurrently, it is crucial to set VMD’s decomposition parameters according to signal characteristics. Inappropriate parameter selection can lead to either excessive or insufficient decomposition. McDonald et al. introduced the maximum correlated kurtosis deconvolution (MCKD) method [11], particularly suitable for processing early bearing fault signals characterized by low signal-to-noise ratios and periodic impact patterns. For composite fault diagnosis, Hong et al. utilized adaptive MCKD to separate fault information from noise-reduced signals [12]. Shen et al. proposed a signal noise-reduction technique based on the Teager energy operator and MCKD [13]. Recent advancements have focused on optimizing the filter length “L” and shift order “M” in MCKD. Lyu et al. optimized these parameters for composite fault diagnosis of gear-tooth wear and bearing outer-ring faults using the quantum genetic algorithm (QGA) [14]. For bearing composite fault diagnosis and prior period estimation, Miao et al. relied on autocorrelation of the envelope signal [15]. Yang et al. adopted permutation entropy as a measurement index to achieve optimal noise-reduction performance and filter length selection for MCKD [16].

Deep learning [17] has garnered increasing attention in the realm of data-driven remaining useful life (RUL) prediction. Deep learning is a subset of machine learning that leverages Artificial Neural Networks (ANNs) with multiple layers to achieve state-of-the-art accuracy in various classification and regression tasks. Unlike traditional machine learning methods, deep learning techniques such as Deep Belief Networks (DBNs) [18], Convolutional Neural Networks (CNNs) [19], and Long Short-Term Memory (LSTM) networks [20] possess the capability to automatically learn hierarchical representations from raw input data without the need for manually crafted rules or domain-specific knowledge. This capacity for powerful representation learning has led to significant successes in various domains, including automatic transmission, speech recognition, natural language understanding, drug discovery, and more.

In the context of data-driven RUL prediction, several studies have explored the application of deep learning: Ren et al. utilized autoencoders to fuse 36 time-domain features [21]. The fused features were subsequently fed into a deep neural network for estimating the RUL of rolling element bearings. Deutsch et al. extracted six time- and frequency-domain features from vibration signals and employed Deep Belief Networks (DBNs) to predict the RUL of spiral bevel gears [22]. Zhu et al. combined wavelet transform with Convolutional Neural Networks (CNNs) for bearing RUL prediction [23]. Wavelet transform was employed to extract time-frequency features, followed by multi-scale CNNs to estimate RUL. Xia et al. applied CNNs to extract robust local features from multi-sensor data and utilized bidirectional LSTM networks to predict the wear depth of cutting tools [24]. These examples illustrate the efficacy of deep learning in capturing intricate patterns and features from complex data, making it a promising approach for enhancing RUL prediction accuracy in various industrial applications.

In this article, we investigate the application of three innovative techniques: maximum correlation kurtosis deconvolution (MCKD) [25], multi-scale permutation entropy (MPE) [26], and long short-term memory (LSTM) recurrent neural network [27]. The objective of this article is to explore the potentials of these techniques for rolling bearings’ RUL prediction, discuss their advantages and limitations, and highlight their contributions to proactive maintenance strategies and asset management. Through a comprehensive review and analysis of existing literature and research studies, we aim to provide insights into the capabilities and practical implications of MCKD, MPE, and LSTM in the context of rolling bearings’ RUL prediction.

The subsequent chapters in this article will delve into the principles, methodologies, applications, and performance evaluations of MCKD, MPE, and LSTM techniques for rolling bearings’ RUL prediction. Comparative analysis and discussion of the advantages and limitations of each technique will be presented, along with potential synergies and future directions for enhanced maintenance practices in rolling element systems.

## 2. Correlation Methods

### 2.1. Maximum Correlation Kurtosis Deconvolution

MCKD is a signal processing technique that aims to enhance the quality and resolution of signals by effectively removing noise and distortion [28]. It is particularly useful in scenarios where the signal of interest is corrupted by additive noise and is convolved with an unknown system impulse response. The mathematical formula can be expressed as follows:

x = h ∗ y(1)

where x is the signal convoluted from various signals on the transmission path, y denotes the impulse signal, and h represents the response of the y signal after passing the transmission path [29].

The core principle of MCKD is to maximize the correlation kurtosis of the deconvolved signal, which is a statistical measure of the signal’s non-Gaussianity. The maximum correlation kurtosis is considered as [30]:

(2) $$O\left(CK_{M}(T)\right)=\frac{\sum_{n=1}^{N}\;{\left(\prod_{m=0}^{M}\;y(n-mT)\right)}^{2}}{{\left(\sum_{n=1}^{N}\;y_{n}^{2}\right)}^{M+1}}$$

By maximizing correlation kurtosis, MCKD aims to enhance the signal’s sparsity and separate it from the noise and distortions.

(3) $${max}_{f^{\prime }}\;CK_{M}(T)={max}_{f^{\prime }}\;\frac{\sum_{i=1}^{N}\;{\left(\prod_{m=0}^{M}\;y_{i-mT}\right)}^{2}}{{\left(\sum_{i=1}^{N}\;y_{i}^{2}\right)}^{M+1}}$$

where *f* represents the filter coefficients of length L. Obtaining the maximum value of the relevant kurtosis is equivalent to solving the following equation where the derivative function is 0.

(4) $$\frac{d}{df_{l}^{\prime }}CK_{M}(T)=0(l=1,2,\cdots ,L)$$

The final coefficients of the filter can be obtained from Equations (1) to (4) and expressed in matrix form:

(5) $$f^{\prime }=\frac{∥y{∥}^{2}}{2\beta^{2}}{\left(X_{0}X_{0}^{T}\right)}^{-1}\sum_{r=T}^{mT}\;X_{r}\psi_{m}$$

where:

(6) $$\beta =\left[\begin{array}{c} y_{1}y_{1-T}\cdots y_{1-MT}\\ y_{2}y_{2-T}\cdots y_{2-MT}\\ \vdots \\ y_{N}y_{N-T}\cdots y_{N-mT} \end{array}\right]$$

(7) $$X_{r}=\left[\begin{array}{ccccc} x_{1-r} & x_{2-r} & x_{3-r} & \cdots & x_{N-r}\\ 0 & x_{1-r} & x_{2-r} & \cdots & x_{N-1-r}\\ 0 & 0 & x_{1-r} & \cdots & x_{N-2-r}\\ \vdots & \vdots & \vdots & \ddots & \vdots \\ 0 & 0 & 0 & \cdots & x_{N-L-r+1} \end{array}\right](r=[T,\cdots ,mT])$$

(8) $$\psi_{m}=\left[\begin{array}{c} y_{1-mT}^{-1}\left(y_{1}^{2}y_{1-T}^{2}\cdots y_{1-MT}^{2}\right)\\ y_{2-mT}^{-1}\left(y_{2}^{2}y_{2-T}^{2}\cdots y_{1-MT}^{2}\right)\\ \vdots \\ y_{N-mT}^{-1}\left(y_{N}^{2}y_{N-T}^{2}\cdots y_{N-MT}^{2}\right) \end{array}\right]$$

The product ${\left(X_{0}X_{0}^{T}\right)}^{-1}$ is well-defined and exists for any matrix $X_{0}$, whether it is square, non-square, full rank, or rank-deficient. This product is not dependent on the matrix being positive definite. The pseudo-inverse allows us to work with a broader class of matrices and it is often used in situations where the standard matrix inverse is not applicable.

In conclusion, the implementation process of the MCKD algorithm can be formulated as follows:

(1) Initialize parameters such as the deconvolution period T, the number of shifts M, and the length of the filter L.
(2) Calculate the $X_{0}^{T}$ and ${\left(X_{0}X_{0}^{T}\right)}^{-1}$ of the input signal x.
(3) Compute the filtered output signal y.
(4) Calculate $\psi_{m}$ and β based on y.
(5) Update the coefficients of the filter f’.

If the kurtosis difference value ΔCK_M_(T) between the signals before and after filtering is smaller than the threshold, end the iteration. Otherwise, repeat steps 3 to 5.

### 2.2. Multi-Scale Permutation Entropy

Multi-Scale Permutation Entropy (MPE) is a powerful tool used for analyzing the complexity and irregularity of time series data [31]. It is particularly useful in the field of signal processing and analysis, where it can provide valuable insights into the underlying dynamics and patterns present in the data.

The core concept of MPE lies in the analysis of the ordinal patterns or permutations that occur within a time series at different scales or resolutions. Ordinal patterns capture the relative order of the data points within a sliding window of fixed length. By examining the frequency and distribution of these ordinal patterns, MPE quantifies the complexity and information content of the time series.

The MPE methodology involves the following steps:

Signal Segmentation: The time series data is divided into non-overlapping segments or windows of fixed length. The length of the window determines the scale or resolution at which the analysis is performed.

(9) $$y_{j}^{\left(s\right)}=\frac{1}{s}\sum_{i=(j-1)s+1}^{js}\;x_{i},j=1,2,\cdots ,[N/s]$$

where s denotes the scale factor and [N/s] denotes the lower integer part.

Ordinal Pattern Generation: Within each segment, the ordinal patterns are generated by assigning a rank to each data point based on its relative position compared to other data points within the window. For example, the smallest data point is assigned rank 1, the second smallest rank 2, and so on [32].

Permutation Encoding: Each ordinal pattern is encoded into a permutation symbol representing the order of ranks. For instance, if the ranks within a window are 3, 1, 2, the corresponding permutation symbol would be 312.

Permutation Frequency Analysis: The frequency of occurrence of each permutation symbol is calculated across all the segments at the given scale. This information reflects the distribution of ordinal patterns and provides insights into the complexity and regularity of the time series.

Entropy Calculation: Entropy is computed based on the probabilities of the permutation symbols. Entropy measures the amount of uncertainty or information content in the time series. Higher entropy values indicate higher complexity and irregularity, while lower entropy values suggest more regular and predictable patterns.

(10) $$H_{p}(m)=-\sum_{r=1}^{R}\;P_{r}lnP_{r}$$

The value of H_p_ reaches its maximum t when $P_{r}$= 1/m!.For convenience, normalization is generally accomplished [33].

(11) $$H_{p}=H_{p}(m)/ln(m!)$$

In the context of rolling bearings’ RUL prediction, MPE can be utilized to analyze the vibration signals obtained from bearings [34]. Vibration signals contain valuable information about the health condition and fault characteristics of the bearings. By applying MPE, the complexity and irregularity of these signals can be quantified, providing useful features for fault diagnosis and RUL prediction.

MPE offers several advantages in RUL prediction analysis. First, it is a non-parametric technique, meaning it does not assume any specific underlying distribution of the data. This flexibility makes it suitable for analyzing complex and non-linear dynamics commonly observed in rolling element systems.

Second, MPE is capable of capturing both short-term and long-term temporal dependencies in the data [35]. By analyzing the ordinal patterns at different scales, MPE can reveal the presence of localized or global patterns, offering a comprehensive understanding of the bearing’s health condition.

Furthermore, MPE can capture subtle changes in the complexity of the vibration signals, allowing for early detection of fault initiation and progression. This early detection can lead to timely maintenance actions and improved RUL prediction accuracy.

By quantifying the complexity and irregularity of vibration signals, MPE-based features can effectively discriminate between different fault conditions and provide valuable information for estimating the remaining operational lifespan of the bearings.

### 2.3. ISSA-LSTM

Long Short-Term Memory (LSTM) is a type of recurrent neural network (RNN) architecture specifically designed to handle the challenges of learning and remembering long-term dependencies in sequential data. Unlike traditional RNNs [36], which suffer from the “vanishing gradient” problem and struggle to capture long-term dependencies, LSTMs are equipped with memory cells and gating mechanisms that enable them to selectively retain and update information over time [37].

The key components of an LSTM network include:

Memory Cell $c_{t}^{*}$: The memory cell serves as the main building block of an LSTM. It maintains an internal state that can be updated or preserved using gating mechanisms. The memory cell enables the LSTM to learn and store information over long sequences [38].

(12) $$c_{t}^{*}=tanh\left(W_{xc}x_{t}+W_{hc}h_{t-1}+b_{c}\right)$$

where $W_{xc}$ represents the connection weight between the input layer and the hidden layer at time t, $W_{hc}$ denotes the connection weight between the hidden layers at time *t* − 1 and t [39], $b_{c}$ and $h_{t-1}$, respectively, represent the biases in the input nodes and the previous time step’s output.

Input Gate $i_{t}$: The input gate determines the amount of new information to be stored in the memory cell at each time step. It takes input from the current time step and the previous hidden state and applies a sigmoid activation function to generate an input gate activation value [40].

(13) $$i_{t}=\sigma \left(W_{xi}x_{t}+W_{hi}h_{t-1}+b_{i}\right)$$

where $W_{xi}$ represents the connection weight between the input layer and the hidden layer at time t, $W_{hi}$ denotes the connection weight between the hidden layers at time *t* − 1 and *t*, $b_{i}$ and $h_{t-1}$, respectively, represent the biases in the input gate and the previous time step’s output, and σ denotes the sigmoid activation function.

Forget Gate $f_{t}$: The forget gate determines the extent to which previous information should be forgotten or preserved in the memory cell. It takes input from the current time step and the previous hidden state and applies a sigmoid activation function to generate a forget gate activation value [41].

(14) $$f_{t}=\sigma \left(W_{xf}x_{t}+W_{hf}h_{t-1}+b_{f}\right)$$

Output Gate: The output gate regulates the amount of information to be output from the memory cell to the next time step. It takes input from the current time step and the previous hidden state and applies a sigmoid activation function to generate an output gate activation value.

(15) $$o_{t}=\sigma \left(W_{xo}x_{t}+W_{ho}h_{t-1}+b_{o}\right)$$

Hidden State: The hidden state carries information from the memory cell and previous hidden state to the next time step. It is computed by applying a tanh activation function to the current input and the memory cell state, and then scaling it by the output gate activation value.

**Figure 1:**
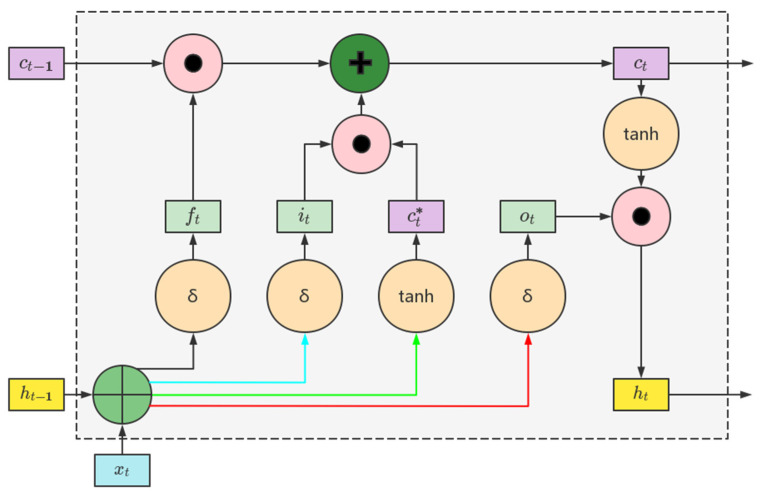
The structure of LSTM.

The structure of LSTM is shown in Figure 1. The use of these gates and memory cells in LSTMs allows the network to selectively update, forget, and output information at each time step, facilitating the learning and retention of long-term dependencies in sequential data.

LSTM has gained significant attention in the field of rolling bearings’ RUL prediction due to its ability to model complex temporal dependencies and effectively handle time-series data. By processing the vibration signals obtained from rolling bearings, LSTM networks can learn the underlying patterns and characteristics indicative of bearing health conditions [42].

In the context of rolling bearings’ RUL prediction, LSTM networks can be utilized after data preprocessing and feature extraction:

LSTM Network Architecture: The LSTM network is constructed with input, hidden, and output layers. The input layer receives the sequence of MPE, which is fed into the LSTM layer. The hidden layer contains the LSTM units responsible for processing and capturing the temporal dependencies in the data. The output layer generates predictions based on the learned patterns and features extracted by the LSTM layer.

Training and Optimization: The LSTM network is trained using a labeled dataset of vibration signals and corresponding RUL values [43]. The network learns to minimize the differences between its predicted RUL values and the actual RUL values. Training involves forward propagation, backpropagation through time, and optimization algorithms such as gradient descent to update the network’s weights and biases.

RUL Prediction: Once the LSTM network is trained, it can be used to predict the remaining useful life of rolling bearings [44]. Given a new sequence of vibration data, the LSTM network processes the sequence through the trained network and generates a predicted RUL value based on the learned temporal patterns and dependencies.

To employ LSTM for remaining useful life prediction, several hyperparameters need to be set in advance, including the number of neurons in the hidden layer, the maximum number of epochs, and the initial learning rate [45]. The predatory and anti-predatory behavior of sparrows in the natural world was the basis for the sparrow search algorithm (SSA). The Sparrow set matrix reads like this:

(16) $$X={\left[x_{1},x_{2}\cdots x_{n}\right]}^{T}\;x_{i}=\left[x_{i,1},x_{i,2}\cdots x_{i,d}\right]$$

In Formula (16), n stands for the total number of sparrows, i equals “1, 2,..., n,” and d refers to the number of dimensions.

The sparrow with a superior position within the population is given priority when it comes to acquiring food, effectively assuming the role of the “finder” responsible for guiding the entire population towards the food source. The update process for determining the finder’s location is as follows:

(17) $$X_{i,j}^{t+1}=\left\{\begin{array}{cc} X_{i,j}^{t}\cdot exp\left(\frac{-i}{\alpha \cdot iter}\right) & R_{2}<ST\\ X_{i,j}^{t}+Q\cdot L & R_{2}⩾ST \end{array}\right.$$

where t denotes the current iteration number, j = (1, 2, …, d); $X_{i,j}^{t}$ represents the position of the ith sparrow in the jth dimension, iter represents the maximum number of iterations, α is a randomly generated number within the range of (0,1), R_2_ (where R_2_ belongs to the interval [0, 1]) represents the danger value, ST (where “ST” belongs to the interval [0.5, 1]) represents the security value, L denotes a 1D matrix, with each element in the matrix being equal to 1, and Q signifies a random integer sampled from a normal distribution with a range of [0, 1]. All individuals, except for the finders, are considered followers. The formula for updating the location of the followers is as follows:

(18) $$X_{i,j}^{t+1}=\left\{\begin{array}{rr} Q\cdot exp\left(\frac{X_{\mathrm{worst}\;}^{t}-X_{i,j}^{t}}{i^{2}}\right) & i>\frac{n}{2}\\ \;X_{p}^{t+1}+\left|X_{i,j}^{t}-X_{p}^{t+1}\right|\cdot A^{+}\cdot L & i\;\leq \;\frac{n}{2} \end{array}\right.$$

where the overall worst position is represented by *X*worst, while A represents 1 × D. A^+^ = A^T^ (AA^T^)^−1^, and 1 or −1 are randomly allocated to each matrix element. When I > n/2, it indicates that the ith follower has a low fitness value, is not fed, and has a very low energy value. It must currently travel to other locations for food to get energy intake.

Improved sparrow search algorithm (ISSA) has been developed to address the challenges faced by SSA when solving engineering optimization problems. SSA is prone to premature convergence, leading to reduced convergence accuracy and local optima. To enhance the algorithm’s performance, ISSA utilizes Tent mapping for population initialization, thereby promoting greater uniformity in the initial population. Chaos initialization introduces randomness, ergodicity, and sensitivity to initial values, which collectively accelerate algorithm convergence. The generation of chaotic sequences based on the Tent map proceeds as follows:

(19) $$T=\left\{\begin{array}{ll} x(n+1)=\mu x(n), & 0⩽x(n)⩽0.5\\ x(n+1)=\mu [1-x(n)], & 0.5<x(n)⩽1 \end{array}\right.$$

Additionally, within the fundamental SSA algorithm, with the progression of iterations, the magnitude of each dimension in the individual sparrow diminishes. Consequently, the search space gradually contracts, elevating the likelihood of getting trapped in local minima. To mitigate this concern, we introduce the sine and cosine algorithm (SCA) into the discoverer location update strategy, accompanied by the integration of a nonlinear sine learning factor. In the initial stages of the search process, this factor proves to be highly valuable, facilitating extensive global exploration. Conversely, during the later phases of the search, it assumes a negligible value, thus contributing to enhanced precision and local refinement capabilities. The improved discoverer location formula and the associated learning factor formula are detailed below:

(20) $$\omega =\omega_{min}+\left(\omega_{max}-\omega_{min}\right)\cdot sin\left(t\pi /\right.i\mathrm{ter}\left.{\;}_{max}\right)$$

(21) $$X_{i,j}^{t+1}\left\{\begin{array}{ll} (1-\omega )\cdot X_{i,j}^{t}+\omega \cdot sin\left(r_{1}\right)\cdot \left|r_{2}\cdot X_{best}-X_{i,j}^{t}\right|, & R_{2}<ST\\ (1-\omega )\cdot X_{i,j}^{t}+\omega \cdot cos\left(r_{1}\right)\cdot \left|r_{2}\cdot X_{best}-X_{i,j}^{t}\right|, & R_{2}⩾ST \end{array}\right.$$

In formula (21), r_1_ is a random number in [0, 2π], and r_2_ is a random number in [0, 2].

To prevent the algorithm from converging prematurely to local optima, we incorporate the Lévy flight strategy into the follower update formula, enhancing its capacity for global exploration. The refined formula is presented below:

(22) $$X_{i,j}^{t+1}=\left\{\begin{array}{c} Q\cdot exp\left(\frac{X_{wort}^{t}-X_{i,j}^{t}}{i^{2}}\right)\;i>\frac{n}{2}\\ X_{p}^{t+1}+X_{p}^{t+1}⊗Levy(d)i\;\leq \;\frac{n}{2} \end{array}\right.$$

**Figure 2:**
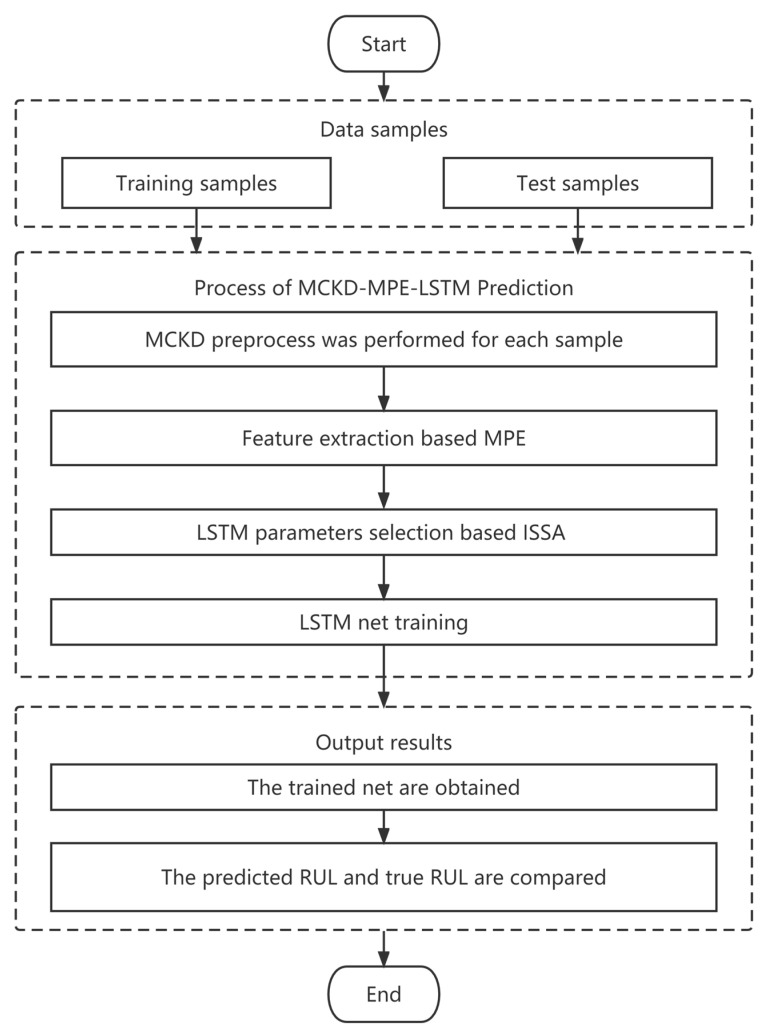
Flowchart of the prediction process.

The flowchart of RUL prediction is shown in Figure 2.

## 3. Experiments and Results

### 3.1. Experimental Platform

To facilitate a comprehensive analysis of the acquired findings, the experimental dataset featuring LDK UER204 rolling element bearings from the XJTU–SY bearing [46] was employed. The dimensional parameters of the bearing are as follows: Inner raceway diameter: 29.3 mm; Outer raceway diameter: 39.8 mm; Bearing’s mean diameter: 34.55 mm; Ball diameter: 7.92 mm; Number of balls: 8; Contact angle: 0°.

There were 15 rolling element bearings of the LDK UER204 type subjected to testing across 3 distinct operating conditions, as indicated in Table 1. The failure modes of the tested bearings include inner race wear, outer race wear, and rolling elements wear.

**Figure 3:**
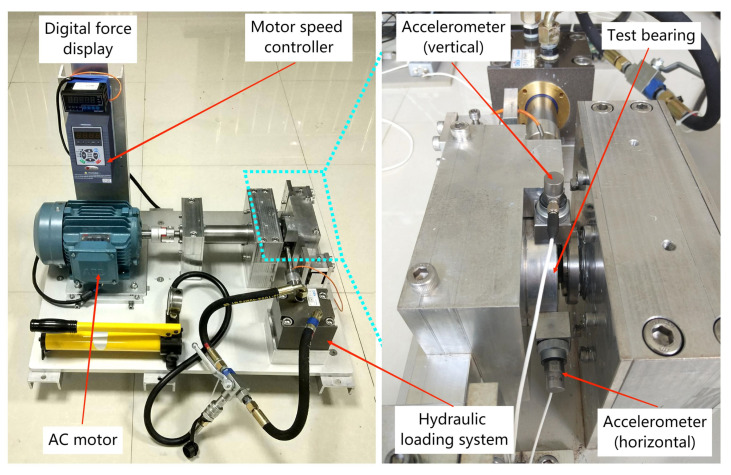
Bearing accelerated life test bed.

Figure 3 provides a visual depiction of the rolling bearings testbed, a sophisticated assembly comprising essential components such as an alternating current (AC) motor, motor speed controller, support shaft, heavy-duty rolling bearings serving as support bearings, and a hydraulic loading system, among others [47]. This experimental platform stands equipped to execute accelerated degradation tests on bearings across varied operational scenarios, simultaneously capturing comprehensive run-to-failure data. The parameters for data acquisition were configured with a sampling frequency of 25.6 kHz and a sampling interval of 1 min. This arrangement resulted in a total of 32,768 individual samples being recorded. Subsequently, the analysis focused on the horizontal vibration signals originating from the dataset designated as “bearing 3_1.” 

**Figure 4:**
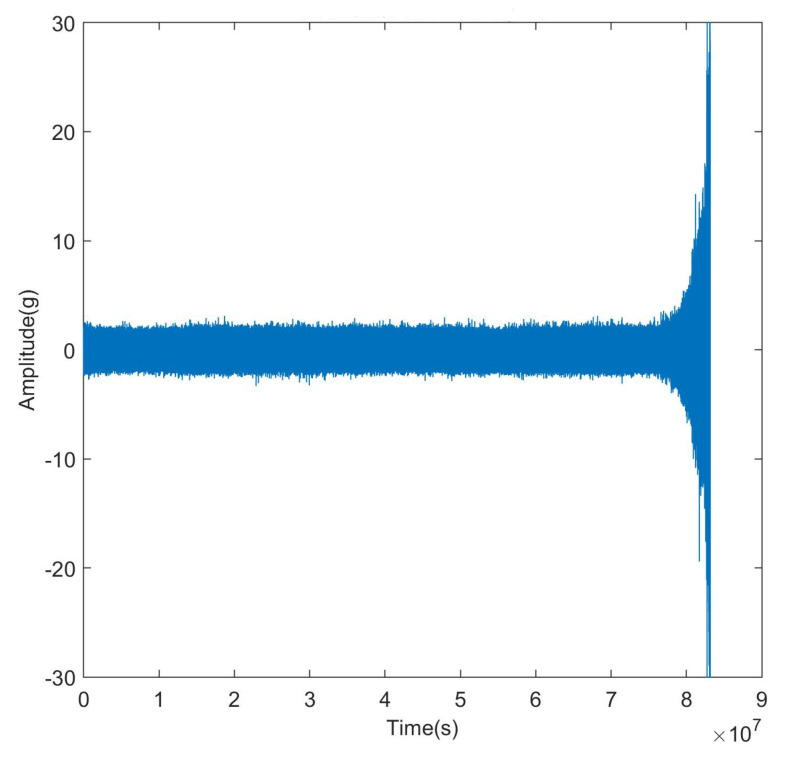
Horizonal vibration signal.

The entire life cycle bearing vibration signal is shown in Figure 4. It is evident from the description that the bearing degradation process comprises two distinct phases: the normal operating stage and the degradation stage. During the normal operating stage, the vibration signals exhibit random fluctuations at a relatively low level. In contrast, the degradation stage is marked by a noticeable increase in the amplitude of vibration signals as a function of operating time. Given this insight, the remaining useful life (RUL) prediction is performed specifically when the bearings enter the degradation stage.

### 3.2. Results and Discussion

**Figure 5:**
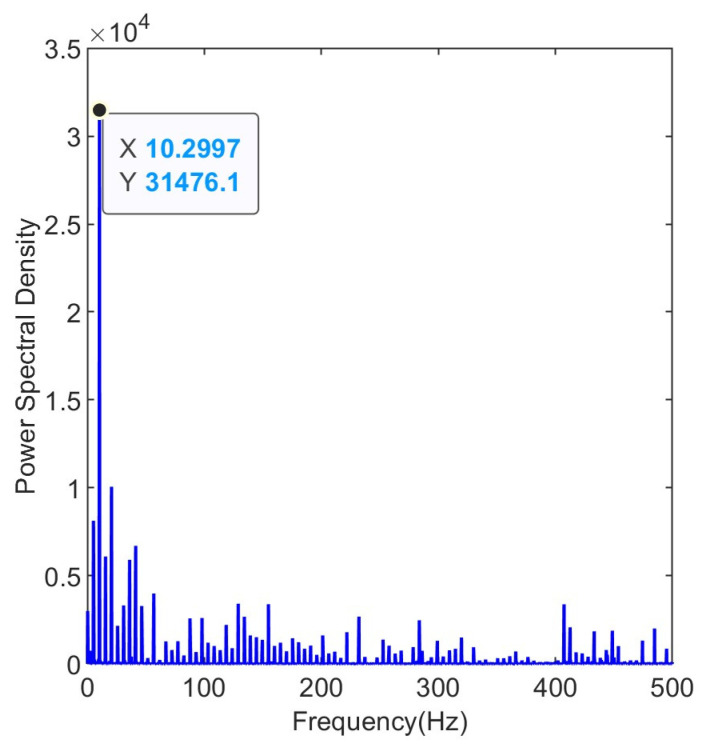
Envelope spectrum of the raw signal.

Due to the inevitable presence of noise in the collected raw vibration signals, the fault frequencies of the bearings are embedded within other spectral components. The envelope spectrum of the raw signal is shown in Figure 5. The envelope spectrum reaches its peak around 10 Hz, which is not the characteristic fault frequency of a rolling bearing. 

Maximum kurtosis deconvolution was performed on the raw vibration signal. The filter length was set to 19. The maximum number of iterations was 30. The deconvolution period was set to 609, which is the ratio between the sampling frequency of 25.6 kHz and the fault frequency of 42.21 Hz. The shift number M was set to 3, indicating that 3 consecutive impacts are considered as a single valid impact. The fault frequency is calculated using Equation (23)

(23) $$f_{o}=\frac{1}{2}Z\left(1-\frac{d}{D}cos\alpha \right)f_{r}$$

where $f_{o}$ is the fault frequency of outer race, Z is the number of balls, d is the inner raceway diameter, D is the outer raceway diameter, α is the contact angle, and $f_{r}$ is the rotation frequency of the shaft.

**Figure 6:**
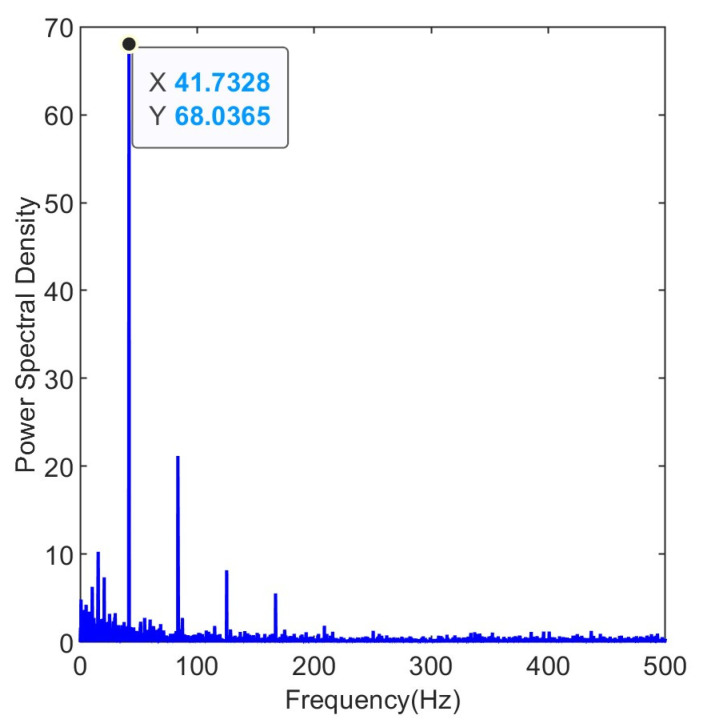
Envelope spectrum of the filtered signal.

The filtered envelope spectrum is shown in Figure 6. The envelope spectrum reaches its peak at 41.73 Hz, which is very close to the fault frequency of 42 Hz. The harmonics are still clearly visible, demonstrating the effectiveness of the maximum kurtosis deconvolution process.

After applying the maximum kurtosis deconvolution for data preprocessing, the multiscale permutation entropy is utilized for feature extraction. The purpose of this is to quantify the degradation information during the operational life of bearings.

Four parameters must be established before MPE can be used [48]: encapsulation dimension m, time series length N, scale factor s, and time delay τ. According to the reference literature, we set the encapsulation dimension m to 5. The time series length N is 3000, which meets the criterion of N ≥ 5 m!. The time delay τ = 1 here since the time delay τ has no significant impact on the outcome [49]. The scale factor s will influence the subsequent feature dimension. When the feature dimension is too small, it may not meet the requirements for RUL prediction. On the other hand, too many features can lead to the curse of dimensionality. Through multiple experiments, we found that setting the scale factor s to 6 yields satisfactory results. Except for the permutation entropy of s = 1, the time series composed of the other five scales of permutation entropy show strong correlation with the remaining useful life time series of the bearing degradation stage, with correlation coefficients reaching above 0.9. Therefore, the scale factor s is set to 6 to obtain permutation entropy at each scale. 

**Figure 7:**
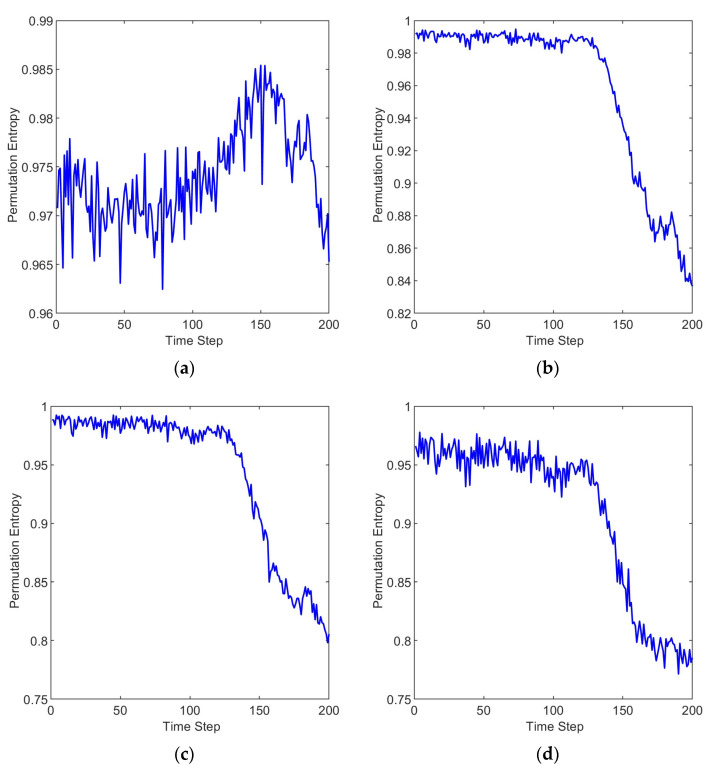

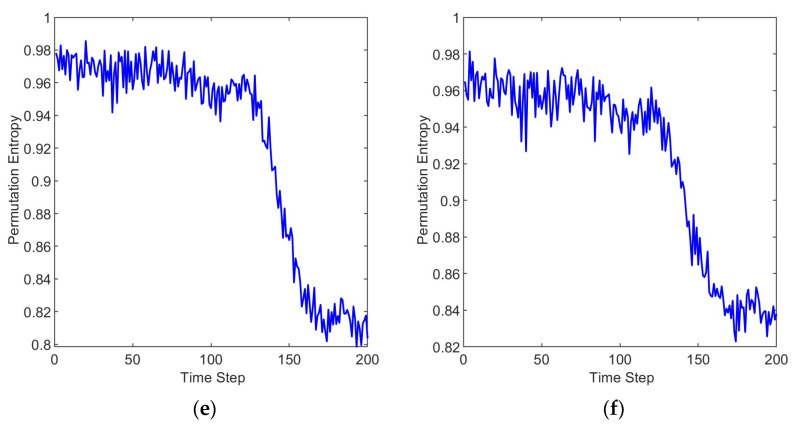
Time evolution curve of permutation entropy. (**a**) s = 1, (**b**) s = 2, (**c**) s = 3, (**d**) s = 4, (**e**) s = 5, (**f**) s = 6.

The time evolution curve of permutation entropy for six scales with bearing degradation is shown in Figure 7. It can be observed that, except for the scale factor s = 1, the permutation entropy values of the other five scales remained relatively stable in the early stages, close to a value of 1. As the early stages of bearing faults emerge, the permutation entropy values gradually decrease. As the bearing faults become more severe, the permutation entropy values decrease to a new steady state. 

The reason why the permutation entropy values decrease as the bearing degrades is that the magnitude of permutation entropy represents the level of disorder in the information. When the bearing is in a healthy state, the vibration signal tends to be more random. As the bearing gradually develops faults, periodic impacts occur due to cyclic collisions at the faulty region. These periodic impacts introduce a more regular pattern in the vibration signal compared to random vibrations. As a result, the permutation entropy values decrease with the bearing degrades.

Furthermore, the permutation entropy with a scale factor s = 1 cannot effectively capture the degradation process of the bearing [50], while the permutation entropy with other scale factors can better accomplish this representation. This is because vibration signals can exhibit rapid variations within continuous time scales, while showing more stable trends over longer time scales. Single-scale permutation entropy might not capture these multi-scale characteristic changes, as it focuses solely on patterns within continuous time scales. Permutation entropy at other scales achieves a coarser representation of the signal across different time scales, mitigating the impact of short-term fluctuations in the signal. This aids in extracting the overall trends present in the signal, resulting in a more comprehensive and accurate feature extraction.

The other permutation entropy values, apart from whose scale factor s = 1, are used as feature vectors to input into the subsequent Long Short-Term Memory (LSTM) neural network model [51]. There are a total of 100 sets of 5-dimensional feature matrices, of which 80 are used as training sets and 20 are used as testing sets. To make more efficient use of computational resources while improving the accuracy of the prediction model, we have truncated the samples to exclude the initial stable operating period. This allows the model to focus more on the later degradation phase where the critical information for prediction lies. 

To employ LSTM for remaining useful life prediction, several hyperparameters need to be set in advance, including the number of neurons in the hidden layer, the maximum number of epochs, and the initial learning rate [45]. Different parameter settings can indeed have a significant impact on the final results obtained. It is crucial to carefully tune these parameters to ensure the best performance and meaningful predictions in specific use case. Experimenting with various parameter combinations and evaluating their effects on the model’s performance is an essential step in optimizing the prediction accuracy. 

Applying the improved sparrow search algorithm mentioned in the reference literature [52] for parameter optimization is a valuable approach. This algorithm can assist in finding optimal or near-optimal parameter settings by simulating the search behavior of sparrows and their interactions within an optimization space. The optimization ranges for the number of neurons in the hidden layer, the maximum number of epochs, and the initial learning rate are [50, 200], [50, 200], and [0.001, 0.1], respectively. The population of sparrows is five, with six iterations. The proportion of discoverers is 0.2 and the warning value is 0.6. It is essential to define the objective function and then use the algorithm to iteratively search for parameter combinations that yield the best results. Selecting the root mean square error (RMSE) as the objective function is a common and appropriate choice. RMSE is a widely used metric in machine learning and prediction tasks to quantify the difference between predicted and actual values, making it suitable for evaluating the performance of remaining useful life prediction model [53]. The goal of the parameter optimization process would be to minimize the RMSE to achieve accurate and reliable predictions. 

**Figure 8:**
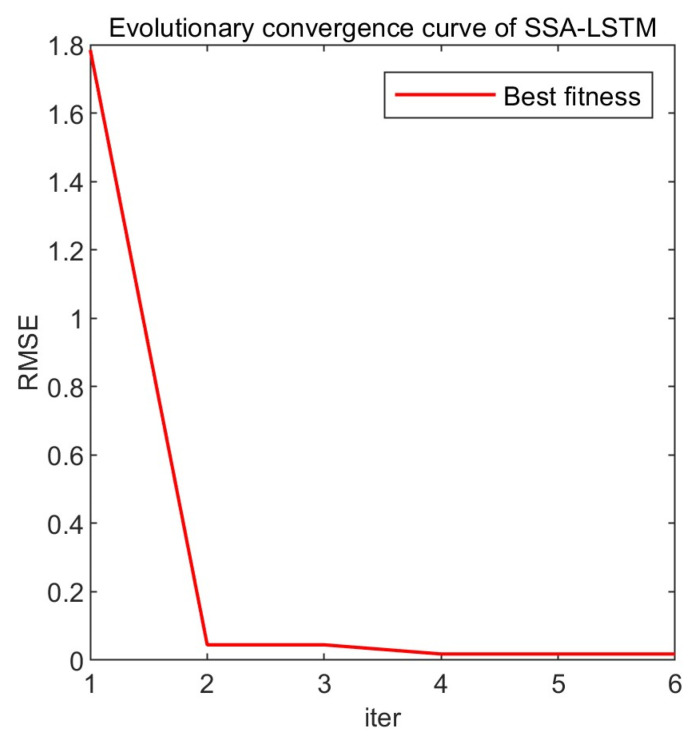
Evolutionary convergence curve of SSA-LSTM.

**Figure 9:**
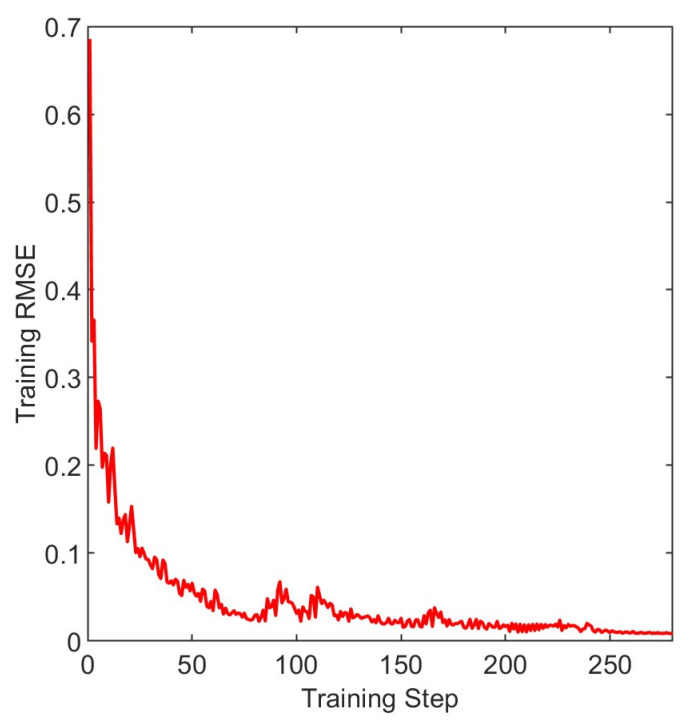
The loss function of the training set.

From Figure 8, it can be seen that the objective function value rapidly decreases during the first and the second iterations and converges by the fourth iteration. The parameter combination obtained through the Sparrow Search Optimization algorithm is as follows: 70, 70, 0.01, which correspond to the number of hidden units, maximum training epochs, and initial learning rate, respectively. The loss function image is shown in Figure 9. The loss function rapidly decreases in the early stage of training and gradually converges smoothly in the later stage.

**Figure 10:**
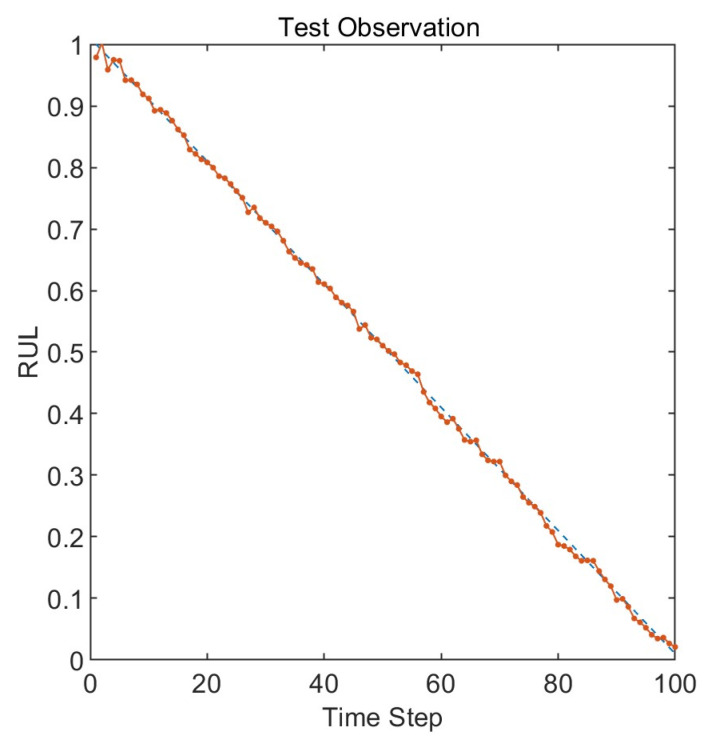
RUL prediction results with optimized parameters.

The remaining useful life prediction results obtained by inputting the feature matrix composed of multiscale permutation entropy into the Long Short-Term Memory neural network are shown in Figure 10. The blue line represents the actual lifespan, while the orange line represents the predicted lifespan. The root mean square error of the prediction results is 0.007. The results indicate that there is a slight drift between the predicted remaining useful life and the actual remaining useful life at the beginning and end, showing a minor endpoint effect. Around time steps 60 and 80, the predicted remaining useful life values are lower than the actual remaining useful life values. The rest of the prediction results are very close to the true values, which validates the effectiveness of the proposed model.

**Figure 11:**
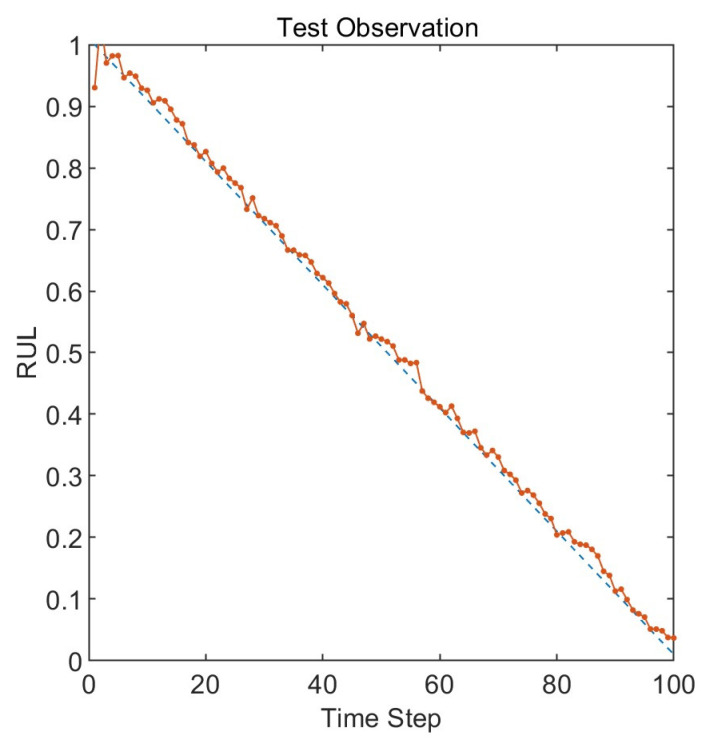
RUL prediction results with default parameters.

The prediction results obtained using the default initial values of 50, 50, and 0.1 for the Long Short-Term Memory neural network are shown in Figure 11. The blue line represents the actual lifespan, while the orange line represents the predicted lifespan. The root mean square error of the prediction results is 0.017. The root mean square error decreased by 58.8% after parameter optimization compared to the default settings. Compared to the optimized model, the results obtained with the default settings show a more pronounced endpoint effect, with a higher degree of deviation from the actual values at time steps 40, 60, and 80.

In order to verify the proposed model, variational modal decomposition (VMD) and support vector machine (SVM) are introduced for comparison in the data preprocessing and life prediction stages, respectively. Root mean square error is selected as the evaluation indicator for the final prediction result, and the specific results are shown in Table 2. When using the same prediction model, the RMSE of using MCKD for data preprocessing is always smaller than the RMSE of using VMD, which indicates that MCKD has better fault information extraction ability than VMD. When using the same data preprocessing method, the RMSE of using LSTM for RUL prediction is always smaller than the RMSE of using SVM, which indicates that LSTM has better predictive performance than SVM.

## 4. Conclusions

In this research, we explored the application of three distinct techniques to predict the remaining useful life (RUL) of rolling bearings: Maximum Correlation Kurtosis Deconvolution (MCKD), Multi-Scale Permutation Entropy (MPE), and Long Short-Term Memory (LSTM) recurrent neural networks. ISSA is employed for configuring parameters, which include the number of neurons in the hidden layer, the maximum number of epochs, and the initial learning rate. Through a comprehensive review of each method, encompassing their underlying principles, methodologies, and real-world applications, we have uncovered novel insights and potential avenues for innovation in the field of predictive maintenance.

(1) Harnessing Unique Strengths: MCKD’s ability to enhance fault signatures, MPE’s prowess in quantifying signal complexity, and LSTM’s proficiency in modeling intricate temporal dynamics represent a trifecta of strengths. By synergistically combining these techniques, we have unlocked a holistic RUL prediction framework that leverages their individual capabilities to provide unprecedented accuracy and reliability.

(2) Towards the Future: Our findings serve as a launching pad for pioneering advancements in rolling element maintenance and asset management. Future research should focus on pushing the boundaries of predictive maintenance by exploring cutting-edge fusion techniques, integrating additional sensor modalities for a more comprehensive view, and delving into innovative feature selection methods. Moreover, the development of user-friendly software tools and frameworks promises to facilitate the seamless adoption of these techniques in diverse industrial settings.

In conclusion, the integration of MCKD, MPE, and LSTM techniques for predicting the RUL of rolling bearings represents a transformative leap in maintenance practices and asset management strategies. By conducting comprehensive analyses and making precise RUL predictions, industries are empowered to execute timely maintenance interventions, optimize performance and reliability, and extend the operational lifespan of rolling bearings. Embracing these techniques not only yields significant cost savings and minimizes downtime but also drives enhanced overall operational efficiency. As we venture forward, the horizon of predictive maintenance holds the promise of even greater innovation and optimization.

## Figures and Tables

**Table 1:** Operating conditions of the tested bearings.

Operating Condition	Radial Force (kN)	Rotating Speed (rpm)	Bearing Dataset
Condition 1	12	2100	Bearing 1–1 Bearing 1–2 Bearing 1–3 Bearing 1–4 Bearing 1–5
Condition 2	11	2250	Bearing 2–1 Bearing 2–2 Bearing 2–3 Bearing 2–4 Bearing 2–5
Condition 3	10	2400	Bearing 3–1 Bearing 3–2 Bearing 3–3 Bearing 3–4 Bearing 3–5

**Table 2:** RMSE of different models.

Method	VMD-SVM	MCKD-SVM	VMD-LSTM	MCKD-LSTM
RMSE	0.023	0.015	0.012	0.007

## Data Availability

http://biaowang.tech/xjtu-sy-bearing-datasets, accessed on 6 September 2023.
